# Enhanced Cytotoxic Activity of Docetaxel-Loaded Silk Fibroin Nanoparticles against Breast Cancer Cells

**DOI:** 10.3390/polym13091416

**Published:** 2021-04-27

**Authors:** Ahmed Al Saqr, Shahid Ud Din Wani, H. V. Gangadharappa, Mohammed F. Aldawsari, El-Sayed Khafagy, Amr S. Abu Lila

**Affiliations:** 1 Department of Pharmaceutics, College of Pharmacy, Prince Sattam Bin Abdulaziz University, Al-kharj 11942, Saudi Arabia; moh.aldawsari@psau.edu.sa (M.F.A.); e.khafagy@psau.edu.sa (E.-S.K.); 2 Department of Pharmaceutics, CT Institute of Pharmaceutical Sciences, Jalandhar 144020, India; shahidpharma2013@gmail.com; 3 Department of Pharmaceutics, JSS College of Pharmacy, JSS Academy of Higher Education and Research, Mysuru 570015, India; hvgangadharappa@jssuni.edu.in; 4 Department of Pharmaceutics and Industrial Pharmacy, Faculty of Pharmacy, Suez Canal University, Ismailia 41522, Egypt; 5 Department of Pharmaceutics and Industrial Pharmacy, Faculty of Pharmacy, Zagazig University, Zagazig 44519, Egypt; a.abulila@uoh.edu.sa; 6 Department of Pharmaceutics, College of Pharmacy, University of Hail, Hail 81442, Saudi Arabia

**Keywords:** breast cancer, cytotoxicity, docetaxel, nanoparticles, silk fibroin

## Abstract

Despite decades of research, breast cancer therapy remains a great challenge. Docetaxel is an antimicrotubule agent that is effectively used for the treatment of breast cancer. However, its clinical use is significantly hampered by its low water solubility and systemic toxicity. The current study was designed to prepare docetaxel (DXL)-loaded silk-fibroin-based nanoparticles (SF-NPs) and to screen their potential antitumor activity against breast cancer cell lines. DXL-loaded SF-NPs were prepared using a nanoprecipitation technique and were evaluated for particle size, zeta potential, entrapment efficiency, and in vitro release profile. In addition, DXL-loaded SF-NPs were screened for in vitro cytotoxicity, cellular uptake, and apoptotic potential against MCF-7 and MDA-MB-231 breast cancer cell lines. The prepared DXL-loaded SF-NPs were 178 to 198 nm in diameter with a net negative surface charge and entrapment efficiency ranging from 56% to 72%. In vitro release studies exhibited a biphasic release profile of DXL from SF-NPs with sustained drug release for 72 h. In vitro cell studies revealed that entrapment of DXL within SF-NPs significantly improved cytotoxic potential against breast cancer cell lines, compared to the free drug, and enhanced cellular uptake of DXL by breast cancer cells. Furthermore, the accumulation in the G2/M phase was significantly higher in cells treated with DXL-loaded SF-NPs than in cells treated with free DXL. Collectively, the superior antitumor activities of DXL-loaded SF-NPs against breast cancer cells, compared to free DXL, could be ascribed to improved apoptosis and cell cycle arrest. Our results highlighted the feasibility of using silk fibroin nanoparticles as a nontoxic biocompatible delivery vehicle for enhanced therapeutic outcomes in breast cancer.

## 1. Introduction

Breast cancer is the most prevalent cancer among women, and it has now surpassed lung cancer as the most commonly diagnosed cancer worldwide [1]. Currently, many therapeutic modalities have been adopted to treat breast cancer, including chemotherapy and targeted therapy as standard treatments, as well as immunotherapy as a promising perspective [2,3,4]. Nevertheless, chemotherapy remains the main core for all therapeutic modalities either to combat cancer progression or to prevent disease recurrence and/or metastasis. Taxanes (paclitaxel or docetaxel) are a commonly used class of chemotherapeutic agents in these contexts [5].

Docetaxel (DXL) is a semisynthetic analog of paclitaxel extracted from the needles of the European yew tree, *Taxus baccata* [6]. It is currently used to treat various types of cancers, including breast cancer, ovarian cancer, prostate cancer, and other human malignancies [7,8]. Docetaxel suppresses tumor growth by various mechanisms such as inhibiting microtubule depolymerization [9], interfering with the expression of apoptotic genes (bcl-2 and bcl-xL) [10], suppressing tumor angiogenesis [11], and/or inhibiting cellular signaling pathways [12]. Clinically, DXL plays a significant role in the prevention of metastatic breast cancer. Nevertheless, because it is lipophilic in nature, the therapeutic efficacy of DXL was adversely compromised by its low systemic bioavailability [8]. One approach to enhance DXT bioavailability was via formulating DXL with high concentrations of polysorbate-80 as a solubilizer and ethanol as a co-solvent. However, these formulations were reported to induce severe side effects including neurotoxicity, hypersensitivity, musculoskeletal toxicity, and hypersensitivity reactions [13,14,15]. In order to circumvent the aforementioned side effects, researchers expended great efforts to develop alternative drug delivery systems such as nanoparticles [16], liposomes [17], polymeric micelles [18], and nanoemulsions [19]. Such formulations have been reported to enhance the therapeutic efficiency of DXL along with improving drug biocompatibility.

Among the various types of drug delivery systems, polymeric nanoparticles have enormous potential as an effective drug delivery system. Polymeric nanoparticles are submicron colloidal particles consisting of different biodegradable materials such as natural or synthetic polymers [20,21]. Polymeric nanoparticles can efficiently carry and deliver both hydrophilic or hydrophobic drugs and imaging agents to targeted sites [20,22]. Polymeric nanoparticles have many advantages in medicine. First, they enable the delivery of active but poorly water-soluble drugs into the biological environment [23]. Second, their structure, shape, and surface properties can be finely tuned to protect the entrapped drug from degradation and/or rapid clearance when incorporated into the biological environment [24]. Most importantly, by virtue of their nano-size range, nanoparticles can preferentially accumulate within tumor tissue via an enhanced permeability and retention (EPR) effect followed by enhanced intercellular uptake/internalization by cancer cells [25]. Accordingly, the major goal in employing nanoparticles as a delivery vehicle is to adjust their particle size and surface properties along with controlling the release of the encapsulated drug in order to attain the precise site of action at a favorable therapeutic concentration [8,9]. 

Silk is a naturally occurring bio-macromolecule extracted from *Bombyx mori* silkworm cocoons, composed mainly of a core filament protein, fibroin [26]. Because of its low immunogenicity and excellent biocompatibility, silk fibroin has been widely used in biomedical applications such as sutures, implantable devices, and tissue engineering [27,28,29]. Recently, silk fibroin has been reported as a promising biopolymer for various drug delivery systems. Several types of silk-fibroin-based drug delivery systems have been introduced in the pharmaceutical field including, films, hydrogels, lyophilized sponges, nanofibers as well as micro- and nanoparticles [27,28,29,30]. SF-based nanoparticles as drug delivery vehicles, in particular, have attracted a lot of interest because of their high drug loading ability, tunable drug release properties, and easy preparation [31,32]. In addition, by virtue of their versatile chemical structure, SF-based nanoparticles hold the ability to encapsulate both hydrophilic and hydrophobic drugs. The hydrophilic amino acid residues in SF, such as serine, glutamate, and aspartic acid, allow the formation of nanoparticles in aqueous solutions, whereas, the hydrophobic amino acid residues, such as alanine, glycine, and tyrosine efficiently facilitate the entrapment of hydrophobic drugs via hydrophobic interaction and π–π stacking [33,34]. Furthermore, by fine-tuning the particle size and surface properties, SF-based nanoparticles may be engineered to increase the therapeutic efficacy of encapsulated drugs along with minimizing the side effect associated with the use of the free drug. Many studies have recently focused on the use of silk-fibroin-based nanoparticles for the delivery of drugs such as cisplatin [35], doxorubicin [36], curcumin [31], paclitaxel [37], and pDNA [38] to various types of cells.

In the present study, therefore, an endeavor has been made to formulate silk-fibroin-based nanoparticles (SF-NPs) loaded with the anticancer drug, docetaxel (DXL). DXL-loaded SF-NPs were prepared by the nanoprecipitation technique. The prepared NPs were characterized, and their in vitro release profile was evaluated. In addition, DXL-loaded SF-NPs were tested for cytotoxicity and apoptosis against breast cancer cell lines. 

## 2. Materials and Methods

### 2.1. Materials

Silk cocoons were procured from the Central Sericultural Research Institute (Mysuru, India). Docetaxel trihydrate (DXL), 3-(4,5-dimethylthiazol-2-yl)-2,5-diphenyl tetrazolium bromide (MTT reagent), and Dulbecco’s modified Eagle medium (DMEM) were procured from Sigma-Aldrich (St. Louis, MO, USA). Dialysis cassette (Slyde-A-Lyzer, 3500 Da MWCO, ThermoFisher, Waltham, MA, USA). All chemicals were of analytical grade.

### 2.2. Cell Lines

A noncancerous epithelial cell line (MCF-10) and two human breast cancer cell lines (MCF-7 and MDA-MB-231) were acquired from the National Center for Cell Sciences (NCCS, Pune, India) and used in passages 60 to 80. All cell lines were certified to be free of mycoplasma contamination. Cultured cells were maintained in Dulbecco’s modified Eagle’s medium (DMEM 90%, Hi-Media, India) supplemented with 10% fetal bovine serum, 100 μg/mL streptomycin, and 100 U/mL penicillin and incubated in 5% CO_2_ atmosphere at 37 °C.

### 2.3. Isolation and Purification of Silk Fibroin

Cocoons from *B. mori* were cut into small pieces and were degummed by boiling with 0.02 M sodium carbonate solution for 30 min. The mixture was allowed to cool at room temperature, washed trice with deionized water, and air dried. The degummed SF was dissolved in 9.3 M lithium bromide at 70 °C for 3 h. To remove salts, dialysis of the resultant solution against deionized water was conducted for 72 h using a Slide-a-Lyzer dialysis cassette (MWCO 3500 Da). The purified SF aqueous solution was then centrifuged for 15 min at 9000 rpm. The concentrated solutions were stored at 4 °C for further study. The final concentration of the aqueous solution of SF was estimated by calculating the weight of the residual solid of a definite volume of solution after drying at 60°C for 48 h [39]. 

### 2.4. Preparation of Docetaxel Trihydrate Loaded in Silk Fibroin Nanoparticles (DXL-Loaded SF-NPs) 

DXL-loaded SF nanoparticles were prepared by a nanoprecipitation technique as described previously [32]. Drug loaded SF-NPs were prepared at different ratios of drug/SF ranging from 1:1 to 1:5 (*w*/*w*) (Table 1). In the nanoprecipitation technique, SF aqueous solution was added dropwise into 5 mL ethanolic solution of DXL with stirring. The suspensions of drug-loaded nanoparticles were maintained for 2 h. Then the drug-loaded nanoparticles were recovered by centrifugation at 12,000 rpm for half an hour. The collected drug loaded SF-NPs were washed twice and were suspended in a saline solution by using an ultrasound processor. SF nanoparticles without drug loading were prepared and served as blanks. The final product was finally lyophilized, without using any cryoprotectant, at 1 × 10^−4^ Torr and −55 °C and the resultant lyophilized NPs were stored at 2–8 °C for further experiments.

To trace the cellular uptake of DXL-loaded SF-NPs, a fluorescent intercalating agent, propidium iodide (PI), was tagged to the surface of the prepared SF-NPs. Briefly, SF-NPs were bath sonicated with an aqueous solution of PI (5 mg/5 mL) for half an hour. The resultant mixture was set for about 24 h at room temperature and then centrifuged at 2500 rpm for 10 min. The supernatant was removed and the solid residue containing PI-tagged NPs was suspended in DMEM for in vitro cell studies.

### 2.5. Evaluation of DXL-Loaded SF-NPs

#### 2.5.1. Determination of Particle Size and Zeta Potential

The mean diameter and surface charge of the prepared SF-NPs loaded with DXL was determined by using a Mastersizer 2000 (Malvern Instruments Ltd., Malvern, Cambridge, UK) at a fixed angle of 90° at 25 °C. All the measurements were performed in triplicate [40].

#### 2.5.2. Scanning Electron Microscopy (SEM) Study

Scanning electron microscopy (SEM) analysis was carried out to inspect the surface morphology of SF-NPs loaded with DXL. Dried samples were positioned on an SEM crust dock and were coated with gold using an ion-sputter coater. The surface morphology of DXL-loaded SF-NPs was visualized using a Hitachi Noran System 7 scanning electron microscope (SEM), at an accelerating voltage of 5 kV and a working distance of 15 mm [40]. 

#### 2.5.3. Determination of Entrapment Efficiency (EE) and Drug Loading (DL)

The amount of drug loaded in the SF-NPs was estimated indirectly by estimating the amount of free, unencapsulated drug in the supernatant of centrifugation. The concentration of DXL in supernatant was analyzed using a high-performance liquid chromatography (HPLC) system (Shimazu, Kyoto, Japan) equipped with a C18 column (250 mm × 40 mm, 5 μm). The mobile phase was composed of acetonitrile and deionized water (55:45, % *v*/*v*). The column temperature was kept at 30 °C and the flow rate was 1 mL/min. The wavelength of UV detection was set at 232 nm. DXL concentration was determined using a preconstructed calibration curve. The entrapment efficiency (EE) and drug loading (DL) were calculated using the following formula:

DL (%) = (Total amount of drug − Amount of unbounded drug)/(Weight of the NPs) × 100(1)

EE (%) = (Total amount of drug − Amount of free drug)/(Total amount of drug) × 100(2)

### 2.6. Structural Characterization of DXL-Loaded SF-NPs

#### 2.6.1. Differential Scanning Calorimetry (DSC)

DXL-loaded SF-NPs were characterized by DSC. The DSC studies were carried out using a DSC 60 (Shimadzu, Tokyo, Japan) for SF, pure DXL, physical mixture (SF + DXL), and DXL-loaded SF-NPs. Samples (5 mg) were placed into flat-bottomed aluminum pans and heated at a fixed rate of 10 °C/min under a nitrogen flow. DSC thermograms were recorded at a temperature range of 25–400 °C, and thermograms were analyzed using TA Universal Analysis software.

#### 2.6.2. Fourier-Transform Infrared Spectroscopy (FTIR) Study

Drug-loaded SF-NPs were characterized by FTIR spectroscopy (Shimadzu-8400, Tokyo, Japan) using the KBr disc pellet technique. The FTIR spectra of pure DXL, SF, physical mixture (SF + DXL), and DXL-loaded SF-NPs were recorded at a resolution of 1 cm^−1^ over the range of 4000–400 cm^−1^. 

#### 2.6.3. X-ray Diffraction Study

X-ray diffraction (XRD) patterns of pure DXL, SF, physical mixture (DXL + SF), and DXL-loaded SF-NPs were attained by using a powder X-ray diffractometer (Rigaku Ultima III, Rigaku Corporation, Tokyo, Japan). The peaks were recorded in a scanning region of 5–45° at a scanning rate of 2°/min.

### 2.7. In Vitro Drug Release Study

The in vitro release behavior of DXL from SF-NPs was studied by the dialysis bag (MWCO 10,000–12,000 Da, Sigma, USA) method. Phosphate-buffered saline of two different pH values, pH 5.5 and pH 7.4, was utilized as the release medium. A definite weight of DXL-loaded SF-NPs was placed in the dialysis bag and was incubated at 37 ± 0.5 °C with 100 mL of release medium under a horizontal shaking speed of 50 rpm. At predetermined time intervals (1, 2, 3, 4, 6, 12, 24, and 48 h), 3 mL aliquot samples were withdrawn from the release media and exchanged with an equal quantity of fresh PBS to maintain sink conditions. The released DXL was quantified by an HPLC system. The experiment was done in triplicate.

### 2.8. Microtiter Tetrazolium (MTT) Assay

MTT assay is a colorimetric assay used for the determination of cell viability [41]. In this study, MCF-7 and MDA-MB-231 cancer cells and noncancerous MCF-10 cells were seeded in 96-well plate at a density of 1 × 10^4^ cells/well and were incubated in 5% CO_2_ atmosphere at 37 °C. At 24 h post-incubation, spent media were exchanged with fresh media containing serial dilutions (25–400 μg/mL) of pure DXL, blank SF-NPs, or DXL-loaded SF-NPs, and the cells were further incubated for 24, 48, or 72 h. At the end of each incubation period, cells were rinsed twice with cold PBS (pH 7.4), and 100 μL of MTT reagent (0.5 mg/mL) was added to each well and further incubated for 4 h. The plates were covered using aluminum foil to avoid exposure to light. The MTT reagent was carefully aspirated and 200 μL of DMSO was added to each well, and the plates were agitated in a gyratory vibrator to improve dissolution of the formed formazan crystals. Finally, to assess cell viability, the optical density was measured using an ELISA microplate reader (ELX-800 BioTek, Midland, ON, Canada) at λ_max_ 570 nm. Untreated cells served as control.

### 2.9. Cellular Uptake Studies

#### 2.9.1. Flow Cytometry Analysis

MCF-7 and MDA-MB-231 cells (2 × 10^5^ cells/well) were plated in a 6-well plate and incubated at 37 °C for 24 h. Cultured cells were treated with 25 μg/mL DXL-loaded SF-NPs tagged with propidium iodide (PI) and incubated at 37 °C. At 24 h post-incubation, cells were rinsed trice with cold PBS, trypsinized for 5 min until complete detachment of the cells. Collected cells were rinsed twice with cold PBS, and cellular uptake of SF-NPs was analyzed using a BD FACSCalibur flow cytometer (BD Biosciences, San Jose, CA, USA). A 670 long-pass filter was used to collect the red fluorescence light of PI. Only viable cells were taken for the analysis.

#### 2.9.2. Confocal Microscopy

MCF-7 and MDA-MB-231 cells were seeded individually in a 6-well plate on a cover-slip (1 × 10^3^ cells/well) pre-coated with poly-L-ornithine and incubated at 37 °C for 24 h. After incubation, the spent medium was decanted, and cultured cells were treated with PI-tagged DXL-loaded SF-NPs and further incubated for 24 h. After the specific incubation time, cells were rinsed twice with cold PBS, cover-slips were collected cautiously without disturbance and positioned on a glass side. The internalization and/or subcellular distribution of DXL-loaded SF-NPs within cancer cells was visualized using an Advanced Spectral Confocal Microscope (Zeiss, LSM 710, Oberkochen, Germany).

### 2.10. Cell Cycle Study

MCF-7 and MDA-MB-231 cells (2 × 10^5^) were seeded in a 6-well plate and were incubated in a CO_2_ incubator for 24 h at 37 °C. Subsequently, spent media was exchanged with fresh media containing either free DXL or DXL-loaded SF-NPs, and the cells were further incubated for 24 h. Cells were rinsed twice with cold PBS, trypsinized, and then harvested and centrifuged for 5 min at 2000 rpm. The supernatant was carefully decanted, and cell pellets were rinsed with PBS, fixed in ice-cold 70% ethanol. The cells were then stained with PI solution consisting of 320 µL propidium iodide and 80 µL ribonuclease A for 30 min in the dark. The stained cells were analyzed by a BD FACSCalibur flow cytometer, and data analysis was done using CytExpert software (Beckman Coulter Life Sciences, Indianapolis, IN, USA).

### 2.11. Cell Apoptosis Analysis

Annexin V-FITC/propidium iodide staining was carried out to detect the mechanism of cell apoptosis upon treatment with DXL-loaded SF-NPs. Briefly, MCF-7 and MDA-MB-231 cells (3 × 10^5^) were seeded in a 6-well plate and were incubated in a CO_2_ atmosphere at 37 °C for 24 h. Cultured cells were treated with either free DXL or DXL-loaded SF-NPs (25 μg/mL) and further incubated for 48 h. At the end of the incubation period, cells were rinsed twice with cold PBS, trypsinized, harvested, and centrifuged. Cell pellets were suspended in annexin-binding buffer and then were stained with annexin V-FIFC conjugate followed by PI solution and incubated at room temperature in the dark for 10 min. Cellular apoptosis/necrosis was finally evaluated using a BD FACSCalibur flow cytometer. Data analysis was conducted with CellQuest Pro software (version 6.0; BD Biosciences). The results are represented as the rate of apoptosis (the percentage of early + late apoptotic cells) [36].

### 2.12. Statistical Analysis 

Statistical analysis was carried out using one-way ANOVA and Student t-test using Graph Pad Prism.

## 3. Results and Discussions

### 3.1. Formulation, Evaluation, and Optimization of DXL-Loaded SF-NPs 

DXL-loaded SF-NPs were fabricated using a nanoprecipitation method. Different formulas were prepared by changing the drug:polymer ratio. Table 1 summarizes the different evaluation parameters of various DXL-loaded SF-NPs formulations. As depicted in Table 1, the particle size of the prepared DXL-loaded SF-NPs ranged from 181.2 ± 4.9 to 198.1 ± 3.9 nm. It was obvious that increasing the drug:polymer ratio from 1:1 to 1:5 resulted in a remarkable increase in the size of the prepared nanoparticles, which might be attributed to the increased viscosity of the polymer solution at higher polymer concentration. 

Zeta potential is a key determinant of colloidal dispersion stability. The zeta potential depicts the degree of electrostatic repulsion in a dispersion between neighboring, similarly charged particles [42]. As shown in Table 1, DXL-loaded SF-NPs possessed zeta potential values greater than −20 mV, indicating good colloidal stability. The net negative surface charge of the prepared nanoparticles was assumed to permit electrostatic repulsion between particles and, thus, ensure the physical stability of nanoparticles upon storage via preventing the SF polymer chains from unrestrained agglomeration. 

The drug loading (DL) and entrapment efficiency (EE) of the DXL-loaded SF-NPs are important parameters for evaluating polymeric nanoparticles. In this study, an indirect method, based on estimating the amount of free, unencapsulated drug in the supernatant of centrifugation, was adopted to calculate DL and EE. Increasing SF concentration was found to increase both drug loading percentage and entrapment efficiency. DL (%) and EE (%) of F5 prepared at drug:polymer ratio of 1:5 were 47.23 ± 2.5% and 72.36 ± 1.6%, respectively, compared to 37.04 ± 0.6% and 56.02 ± 2.1 for F1 prepared at a drug:polymer ratio of 1:1. The increase in nanoparticle entrapment efficiency might be ascribed to the increased ability of SF polymers to entrap higher amounts of DXL upon increasing polymer concentration. Of note, recent approaches have been adopted for direct and accurate measurement of the encapsulation efficiency and drug loading of highly hydrophobic molecules, loaded in silk fibroin NPs [43,44]. Such methods could offer the chance for obtaining a future clinical-grade product.

On the basis of different evaluation parameters, DXL-loaded SF-NPs (F5) that showed the highest entrapment efficiency and drug loading while maintaining an appropriate particle size (<200 nm) were selected for further investigations.

### 3.2. Characterization of Optimized DXL-Loaded SF-NPs

#### 3.2.1. SEM Study

**Figure 1:**
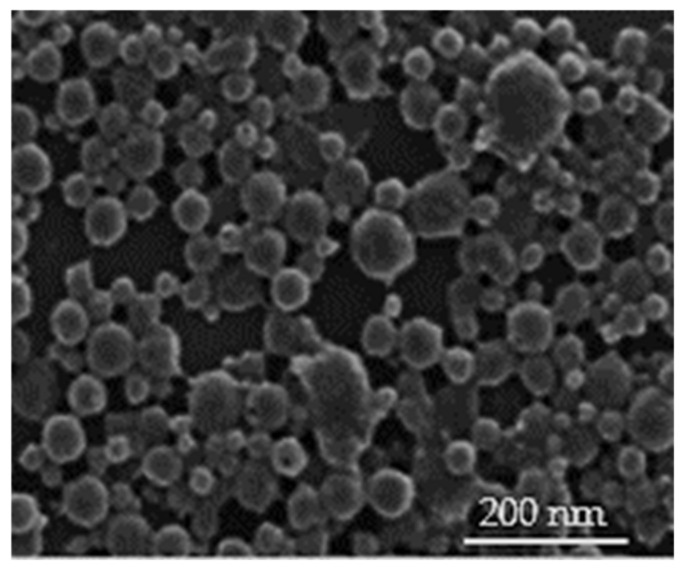
Scanning electron microscopy (SEM) picture of DXL-loaded SF-NPs (magnification ×100,000).

The surface morphology of optimized DXL-loaded SF-NPs was scanned using scanning electron microscopy (SEM). As shown in Figure 1, DXL-loaded SF-NPs had a rounded shape with a smooth surface. Of note, SEM images showed a smaller size of DXL-loaded SF-NPs compared with dynamic light scattering (DLS) results. It is well known that SEM determines the size of the dried sample, whilst DLS estimates the hydrodynamic diameter of the particle core along with the solvent sheath attached to the particle. Consequently, the size observed on SEM images is always smaller than that determined by DLS [40].

#### 3.2.2. FTIR Analysis of the Nanoparticles

**Figure 2:**
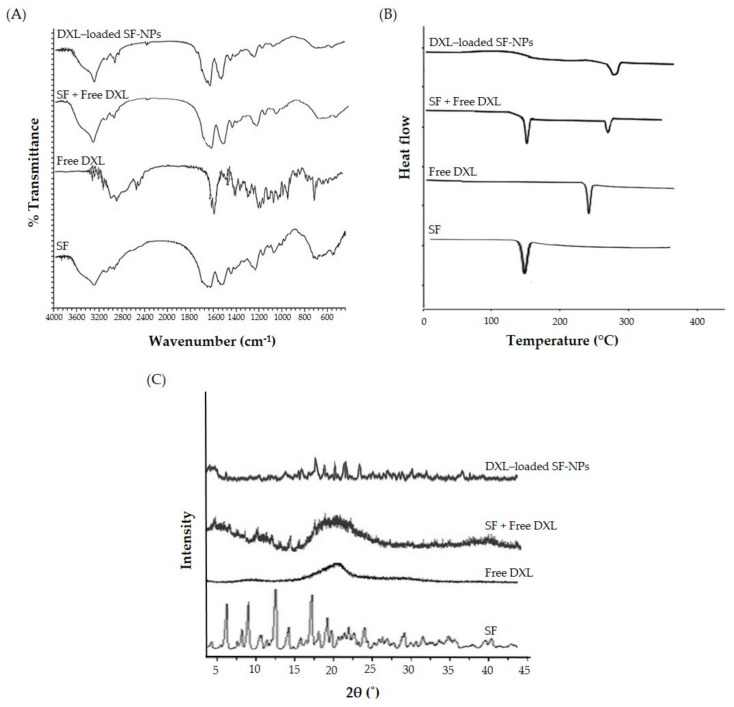
Physicochemical characterization of DXL-loaded SF-NPs. (**A**) Fourier-transform infrared spectra, (**B**) differential scanning calorimetry thermograms, and (**C**) X-ray diffraction spectra.

The interactions, if any, between the drug (DXL) and the polymer (SF) were explored by FTIR studies (Figure 2A). FTIR spectra of DXL showed characteristic peaks at 3368 cm^−1^ (N–H and O–H, stretching), 2984 cm^−1^ (C–H, stretching), 1738 cm^−1^ (C=O, stretching), and 1492 cm^−1^ (C=C, stretching). Typical bands of crystalline β-sheet domains were observed in the spectra of SF, demonstrating that silk fibroin was in its stable conformation. In particular, we identified the bands related to amide I, amide II, and amide III at about 1623, 1539, and 1232 cm^−1^, respectively. The FTIR spectrum of a physical mixture of (DXL + SF) preserved the characteristics the C=O stretching, N–H stretching, and O–H stretching peaks of DXL. Of interest, FTIR spectrum of optimized DXL-loaded SF-NPs retained the characteristic absorption peaks of both free DXL and SF. In addition, no major shift in the absorption peaks was observed upon encapsulation of DXL within SF-NPs, suggesting the compatibility of DXL with SF.

#### 3.2.3. Differential Scanning Calorimetry (DSC) Measurement

DSC is usually adopted to gain information about the physical properties of drugs, polymers, and formulations. It measures the heat gain or loss in samples as a function of temperature [45]. The DSC thermograms of free DXL, SF, physical mixture, and optimized DXL-loaded SF-NPs are shown in Figure 2B. Pure DXL showed a characteristic endothermic peak at 165 °C. SF showed a melting endothermic peak at 286 °C, which was attributed to the thermal degradation of silk fibroin chains. The DSC thermogram of a physical mixture (DXL + SF) showed the characteristic peaks of individual components, indicating the compatibility between SF and DXL. Of interest, the endothermic peak of DXL at 165 °C, which was observed in the thermograms of pure DXL and physical mixture, was not observed in DXL-loaded SF-NPs. The disappearance of the endothermic peak related to DXL in DXL-loaded SF-NPs indicates the dispersion and/or entrapment of DXL molecules in the polymeric network of SF.

#### 3.2.4. X-ray Diffraction (XRD) Measurement

XRD analysis was conducted to scrutinize the interaction between DXL and SF and the degree of sample crystallinity. The X-ray difractogram of pure DXL showed characteristic peaks at 2θ angle of 8.9°, 14.2°, 17.4°, and 19.6°, revealing the crystalline nature of the drug. On the other hand, no sharp peaks were observed in the difractogram of DXL-loaded SF-NPs (Figure 2C). These findings suggest the existence of DXL in a disordered crystalline/amorphous form within the polymeric matrix or its presence in the form of molecular dispersion [46].

### 3.3. In Vitro Docetaxel Trihydrate Release from DXL-Loaded SF-NPs

**Figure 3:**
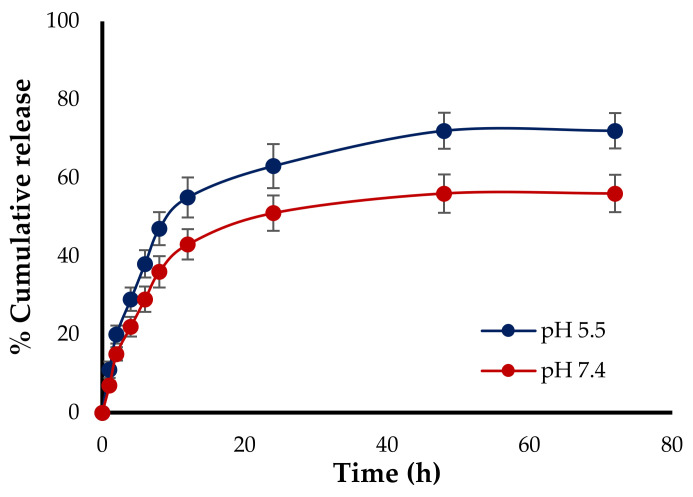
In vitro release study of DXL from SF-NPs in phosphate-buffered saline (pH 5.5 and 7.4).

Controlled-release drug delivery systems are generally designed to grant the release of definite amounts of entrapped drug at a specific site of action over a predetermined time span. Consequently, the potential of SF-NPs to control and/or sustain the release of the entrapped DXL was assessed. The in vitro release profile of DXL from drug-loaded SF-NPs was studied in phosphate-buffered saline (PBS) of two different pH values, pH 5.5 and 7.4, in order to mimic the acidic environment within tumor tissue and physiological pH, respectively. The cumulative drug release percentage was plotted against time, and the amount of drug released from SF-NPs was quantified for a period of 72 h using the HPLC method. As shown in Figure 3, the cumulative release percentage of DXL from SF-NPs was significantly higher (63.25 ± 5.6%) in PBS pH 5.5 compared to that in PBS pH 7.4 (51.18 ± 4.5%) within 24 h, followed by sustained drug release for the next 48 h at both pH values. The remarkably higher drug release from SF-NPs at acidic pH could be ascribed to the basic nature of the entrapped drug. In addition, the in vitro release of DXL from SF-NPs at both buffers showed a biphasic release pattern; consisting of an initial burst of drug release followed by a prolonged release for up to 72 h. The initial burst effect might be ascribed to the quick release of drug molecules adsorbed at the surface of the NPs and/or entrapped near the surface. On the other hand, the prolonged release of DXL from SF-NPs might be ascribed to the slow diffusion of the drug through the polymeric matrix of β-sheets of SF-NPs. This bi-phasic release is desirable in cancer therapy as the early drug release during the burst stage would directly restrain the cell growth immediately after administration, while the prolonged release of drug would continue to repress tumor growth for a prolonged period. Furthermore, the higher drug release at acidic pH affirms the improved utility of SF-NPs for delivering entrapped drugs to the acidic tumor microenvironment. 

### 3.4. In Vitro Cytotoxicity

**Figure 4:**
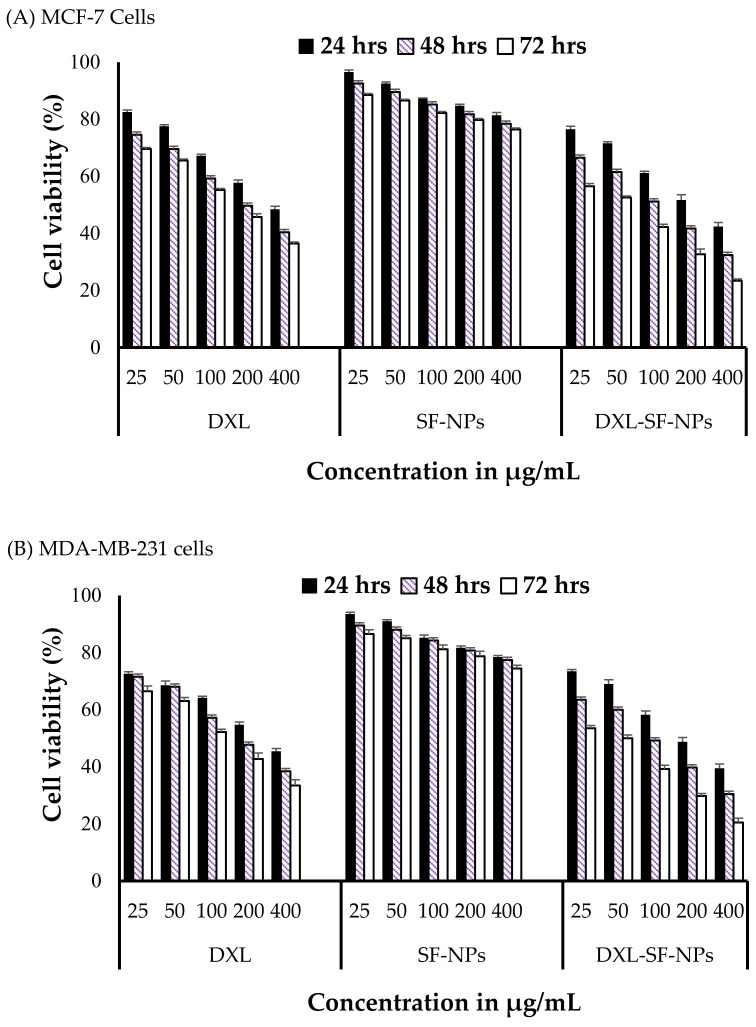
In vitro cytotoxicity of DXL-loaded SF-NPs against breast cancer cell lines. (**A**) MCF-7 and (**B**) MDAMB-231 cells were incubated with different concentrations (25–400 μg/mL) of free DXL, SF-NPs, or DXL-loaded SF-NPs for different incubation times (24, 48, and 72 h), and cell viability was then assessed by MTT assay. Data represent mean ± SD.

To assess the cytotoxicity of free DXL and DXL-loaded SF-NPs, an MTT assay was conducted using breast cancer cell lines (MCF-7 and MDA-MB-231) and a noncancerous epithelial cell line (MCF-10). Cells were incubated with serial concentrations (25−400 μg/mL) of free DXL or DXL-loaded SF-NPs for 24, 48, and 72 h. The cytotoxicity of blank (drug-free) SF-NPs was also studied to nullify any nonspecific effect. Blank SF-NPs did not induce evident cytotoxicity against tested cell lines; with more than 85% of treated cells remaining viable even after incubation for 72 h. On the contrary, both free DXL and DXL-loaded SF-NPs exerted potent cytotoxic effect against MCF-7 (Figure 4A) and MDA-MB-231(Figure 4B) breast cancer cells in a concentration- and time-dependent manner. Nevertheless, DXL-loaded SF-NPs showed superior cytotoxic effect against both breast cancer cell lines compared to free DXL. The IC_50_ values of DXL-loaded SF-NPs against MCF-7 and MDA-MB-231 cells were 49.76 and 59.16 µg/mL, respectively, compared to 56.07 and 65.23 µg/mL for free DXL after a 24 h incubation time. These results are consistent with previous reports that emphasized the increased toxicity of plain drug upon its encapsulation within a nanoparticulate system [7,13,47]. The proposed mechanism for the enhanced cytotoxicity of DXL-loaded SF-NPs might be ascribed to the preferential cellar uptake of SF-NPs with subsequent efficient drug release within tumor cells. Of note, contrary to free DXL, DXL-loaded SF-NPs did not exert remarkable cytotoxicity against noncancerous epithelial cells (MCF-10); cell viability was >85% even upon treatment with 400 μg/mL for 24 h (data not shown). Similar findings were reported by Montalbán et al. [31], who revealed that the cytotoxic effect of curcumin-loaded SF-NPs was selective against hepatocellular carcinoma (Hep3B) cells; with not evident toxicity against normal human bone marrow–derived mesenchymal stem cells (hBMSCs) cells. Our results, collectively, suggest that entrapment of DXL within SF-NPs not only enhances the cytotoxic potential of the free drug against cancerous cells but also spares normal cells from the cytotoxic effect of the free drug.

### 3.5. In Vitro Cellular Uptake Studies of Nanoparticles

**Figure 5:**
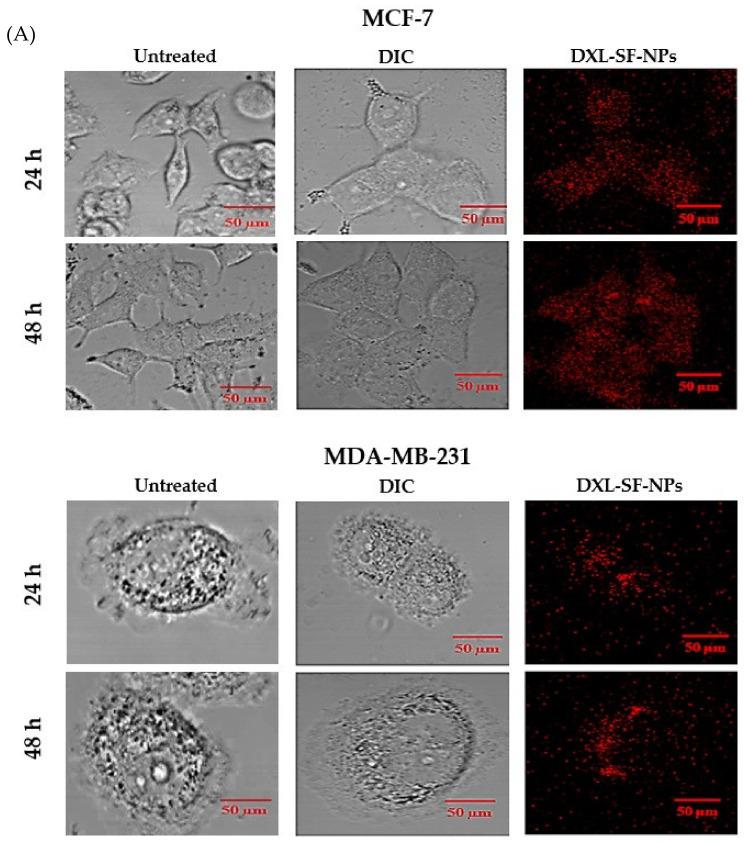

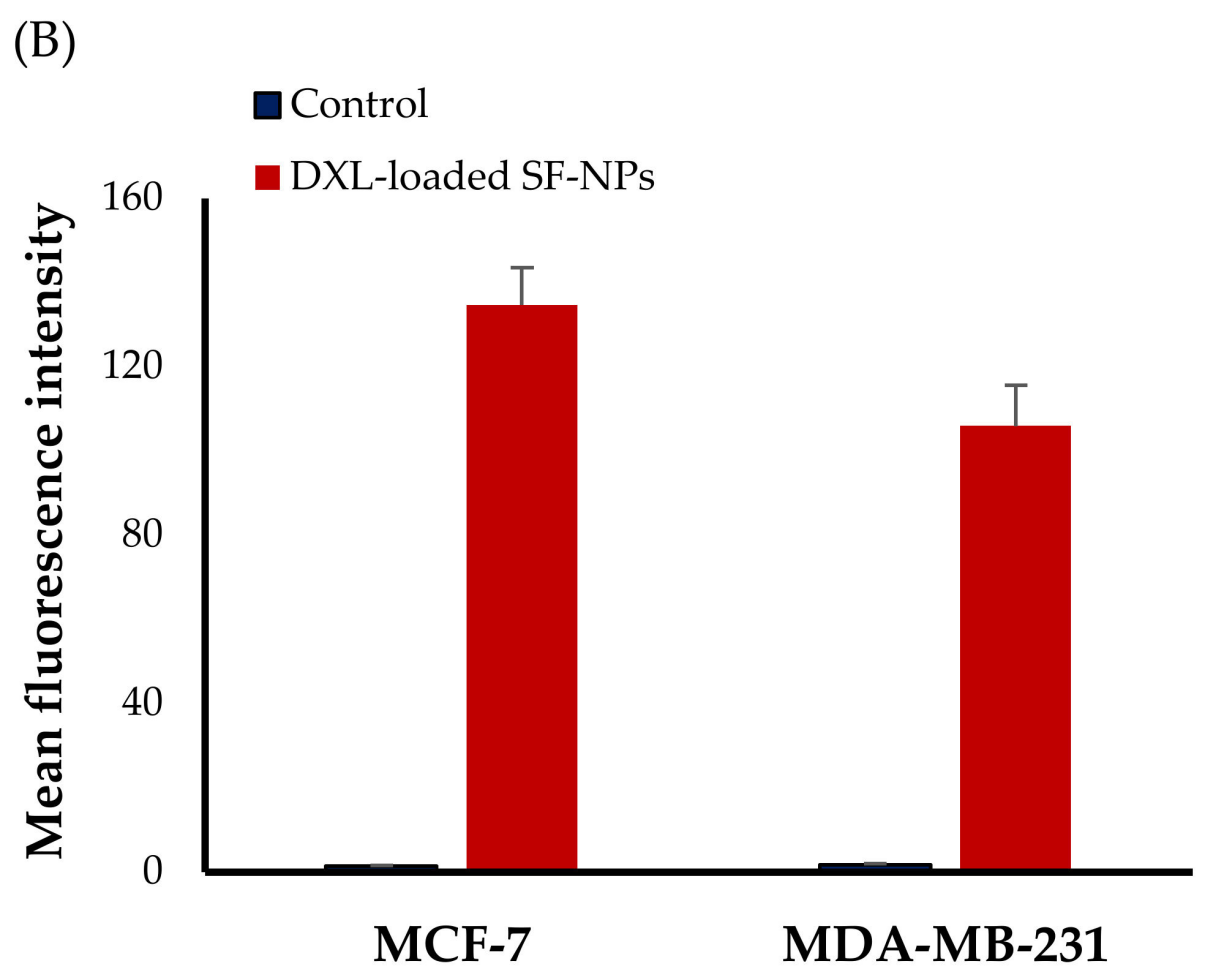
(**A**) Qualitative cellular uptake study of DXL-loaded SF-NPs by MCF-7 and MDA-MB-231 breast cancer cells by confocal microscopy analysis. DIC refers to differential interference contrast image. (**B**) Quantitative assessment of cellular uptake of DXL-loaded SF-NPs by MCF-7 and MDA-MB-231 breast cancer cells by flow cytometric analysis.

Therapeutic efficiency against cancer depends mainly on the ability of the therapeutic agents to reach their distinct targets. Nanoparticles have been widely used to enhance the cellular delivery of entrapped drugs to target sites. Accordingly, cellular uptake of DXL-loaded SF-NPs by breast cancer cells was investigated. DXL-loaded SF-NPs were fluorescently labeled with the red-fluorescent propidium iodide (PI) dye, and cellular uptake of DXL-loaded SF-NPs by MCF-7 and MDA-MB-231 breast cancer cells was assessed quantitatively and qualitatively. Figure 5A depicts the cellular uptake of DXL-loaded SF-NPs by both MCF-7 and MDA-MB-231 cells, as visualized by confocal microscopy. No red fluorescence signals were observed in the control cells. In contrast, both cancer cell lines incubated with DXL-loaded SF-NPs showed strong fluorescence intensities, suggesting that DXL-loaded SF-NPs were efficiently taken up by both cell lines. In addition, confocal fluorescence microscopy studies revealed a time-dependent uptake of DXL-loaded SF-NPs by breast cancer cell lines (MCF-7 and MDA-MB-231); with higher fluorescence intensities observed at 48 h post incubation with DXL-loaded SF-NPs. Of note, MCF-7 showed higher fluorescence signals, compared to MDA-MB-231 cells, signifying preferential uptake of DXL-loaded SF-NPs.

Next, to quantitatively evaluate the cellular uptake of DXL-loaded SF-NPs by breast cancer cells, MCF-7 and MDA-MB-231 cells were incubated with PI-labeled DXL-loaded SF-NPs for 24 h, and the mean intracellular fluorescence intensities (MFIs) were measured by using flow cytometry. As shown in Figure 5B, DXL-loaded SF-NPs were efficiently taken up by both cancer cells. However, higher MFI was observed in MCF-7 cells, compared to that in MDA-MB-231 cells, suggesting preferential uptake of DXL-loaded SF-NPs by MCF-7 cells. Such preferential uptake of SF-NPs by MCF-7 cells might explain the superior cytotoxic potential of DXL-loaded SF-NPs observed in MCF-7, compared to that in MDA-MB-231 cells.

### 3.6. Cell Cycle Analysis

**Figure 6:**
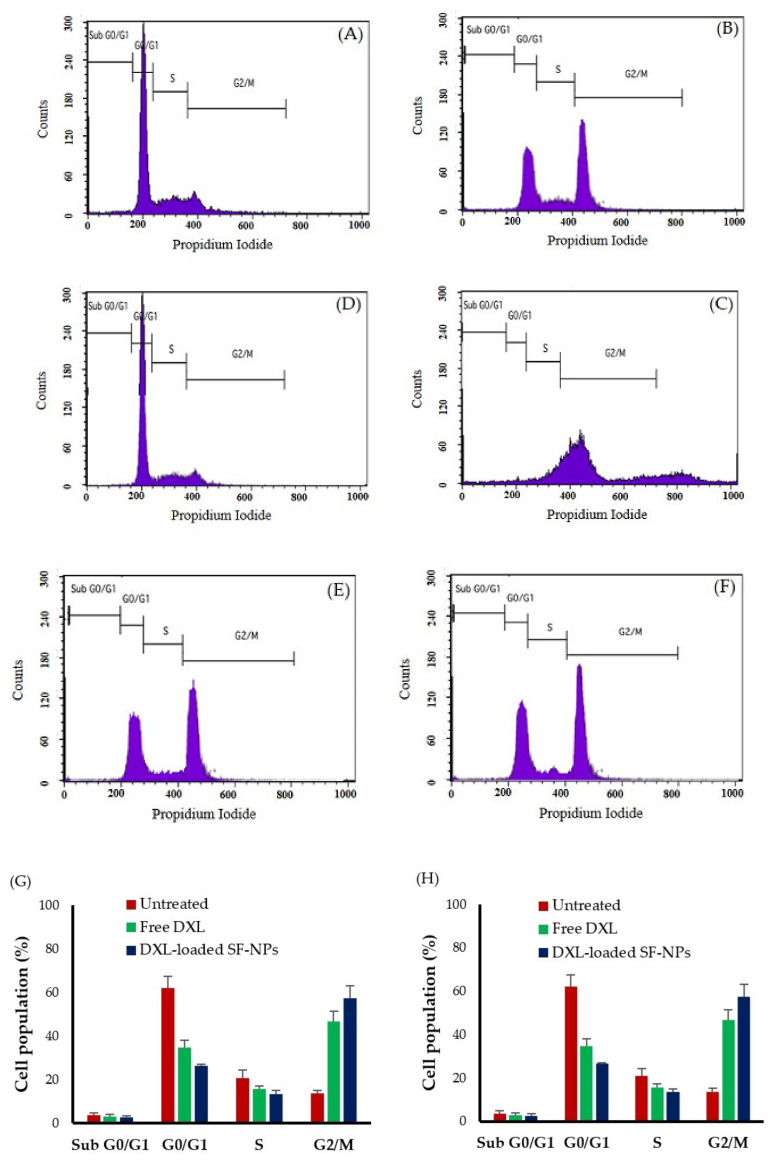
Effect of free DXL and DXL-loaded SF-NPs on cell cycle progression. Flow cytometric analysis of cell populations at different cell cycle stages of (**A**) untreated MCF-7 cells, (**B**) MCF-7 cells treated with free DXL, (**C**) MCF-7 cells treated with DXL-loaded SF-NPs, (**D**) untreated MDA-MB-231 cells, (**E**) MDA-MB-231 cells treated with free DXL, (**F**) MDA-MB-231 cells treated with DXL-loaded SF-NPs. Analysis of the cell cycle of (**G**) MCF-7 cells or (**H**) MDA-MB-231 cells treated with either free DXL or DXL-loaded SF-NP. Data represent mean ± SD.

Cell cycle arrest and induction of apoptosis are two primary causes for the inhibition of cell proliferation. Docetaxel has been reported to target microtubules, resulting in cell cycle progression arrest at mitosis and subsequently leading to cell death. To establish whether DXL-loaded SF-NPs affect cell cycle progression, the distribution of either MCF-7 or MDA-MB-231 cancer cells in various cell cycle stages upon treatment with free DXL or DXL-loaded SF-NPs was determined using flow cytometry (Figure 6). Consistent with previous reports [48,49,50], treatment with free DXL for 24 h efficiently mediated G2/M cell cycle arrest in MCF-7 and MDA-MB-231 breast cancer cells, whereas the number of cells in G0/G1 phase decreased compared with the untreated cells (Figure 6). Of interest, DXL-loaded SF-NPs resulted in G2/M phase cell cycle arrest, which was superior to that caused by free DXL. 

The accumulation of the cells in the G2/M phase was 68.49% and 57.51% in MCF-7 and MDA-MB-231, respectively, upon treatment with DXL-loaded SF-NPs, compared to 49.63% and 46.78% in MCF-7 and MDA-MB-231 cells treated with free DXL. These results suggest that, following cellular uptake/internalization of SF-NPs, DXL was released efficiently in the cytosol resulting in cell cycle arrest in the G2/M phase. These findings are in accordance with the results of the cytotoxicity assay and could provide further evidence promoting the therapeutic application of DXL-loaded SF-NPs in cancer therapy.

### 3.7. Cell Apoptosis Study

**Figure 7:**
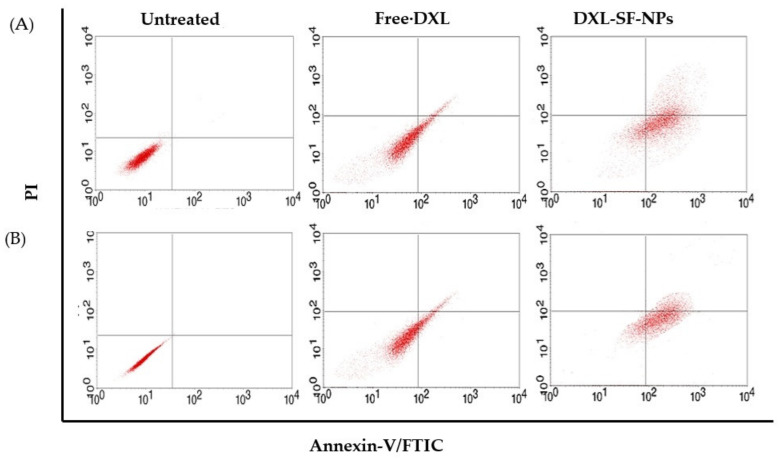

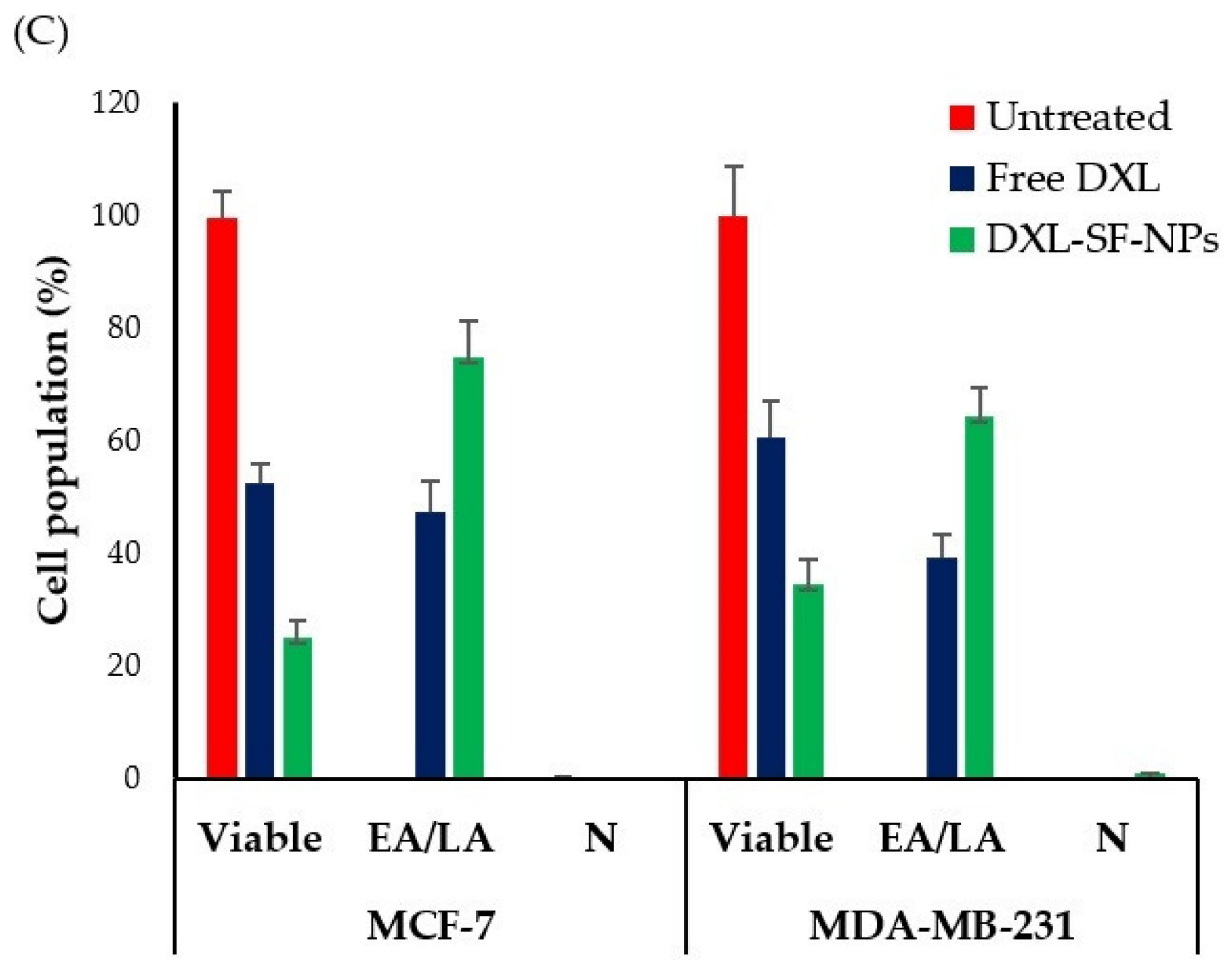
Flow cytometry analysis data from annexin V-FITC staining. Dot plots representing the distribution of apoptotic cells of (**A**) MCF-7 cells and (**B**) MDA-MB-231 cells following staining with annexin V-FITC and propidium iodide. (**C**) Quantitative analysis of the percentage of apoptotic cells upon treatment with either free DXL or DXL-loaded SF-NPs.

Apoptosis is considered the major cell death mechanism in response to taxanes [46]. In this study, breast cancer cells were stained with annexin V-FITC and propidium iodide and analyzed by flow cytometry. Annexin V staining could efficiently differentiate viable cells (stained negative for both PI and annexin V-FITC; lower left quadrant), early apoptotic cells (stained negative for PI and positive for and annexin V-FITC; lower right quadrant), late apoptotic cells (stained positive for both PI and annexin V-FITC; upper right quadrant), and necrotic cells (stained positive for PI; upper left quadrant). As depicted in Figure 7, the rate of apoptosis (the sum percentage of early and late apoptotic cells) in untreated MCF-7 and MDA-MB-231 cells was 0.08% and 0.06%, respectively. Of interest, DXL-loaded SF-NPs induced a potent apoptotic response against both cell lines, compared to free DXL. The apoptotic rates of DXL-loaded SF-NPs were 74.74 ± 4.6% and 64.33 ± 3.8% in MCF-7 and MDA-MB-231 cells, respectively, compared to 47.48 ± 3.5% and 39.28 ± 3.1% for MCF-7 and MDA-MB-231 cells treated with free DXL. These results suggest that encapsulation of DXL within SF-NPs could efficiently potentiate the cytotoxic efficacy of DXL not only via enhancing the cellular uptake of the drug by breast cancer cells but also by promoting significant cell cycle arrest with subsequent induction of potent apoptotic response as well. 

## 4. Conclusions

In the present study, we successfully prepared DXL-loaded SF-NPs using the nanoprecipitation method. The prepared DXL-loaded SF-NPs were spherical in shape, showing uniform size distribution and high entrapment efficiency. In addition, cell culture studies proposed that entrapment of DXL within SF-NPs efficiently enhanced the cellular uptake of DXL by breast cancer cells and exerted superior cytotoxicity, compared to free DXL. Furthermore, cell cycle analysis confirmed that the antitumor activity of DXL-loaded SF-NPs is mediated mainly via arresting the G2/M phase with the subsequent induction of cellular apoptosis. To sum up, our results suggest that DXL-loaded SF-NPs might be considered as a promising biocompatible formulation to be adopted for the treatment of breast cancer. Nevertheless, further studies are urgently needed to support our observations of the antitumor potential of silk fibroin nanoparticles in vivo as well as to emphasize the feasibility of large-scale production of silk fibroin nanoparticles while maintaining good manufacturing practice (GMP) regulations.

## Figures and Tables

**Table 1:** Characterization of DXL-loaded SF-NPs prepared at different drug: SF polymer ratios.

Formula	Drug: Polymer Ratio (*w*/*w*)	Particle Size (nm)	Zeta Potential (mV)	Entrapment Efficiency (%)	Drug Loading (%)
F0	0:1	178.1 ± 6.2	− 18.6 ± 1.1	0.00 ± 0.0	0.00 ± 0.0
F1	1:1	181.2 ± 4.9	−23.9 ± 0.9	56.02 ± 2.1	37.04 ± 0.6
F2	1:2	182.5 ± 3.6	−24.2 ± 1.3	57.23 ± 1.5	38.04 ± 2.4
F3	1:3	184.1 ± 5.2	−24.7 ± 1.1	66.06 ± 3.4	41.23 ± 1.3
F4	1:4	186.2 ± 4.4	−24.9 ± 1.2	69.34 ± 2.7	43.63 ± 1.4
F5	1:5	198.1 ± 3.9	−26.6 ± 0.8	72.36 ± 1.6	47.23 ± 2.5

Data represent mean ± SD of three independent experiments.

## Data Availability

The data presented in this study are available on request from the corresponding author.
